# Assessment of salivary alpha amylase and mucin-4 before and after non-surgical treatment of peri-implant mucositis

**DOI:** 10.1186/s40729-022-00429-z

**Published:** 2022-07-14

**Authors:** Hajer A. Aldulaijan, Abeer S. Al-Zawawi, Marwa Y. Shaheen, Dena Ali, Darshan Devang Divakar, Amani M. Basudan

**Affiliations:** 1 Department of Periodontics and Community Dentistry, College of Dentistry, King Saud University, Riyadh, 11451 Saudi Arabia; 2 Department of General Dental Practice, Kuwait University, P. O. Box 24923, 13110 Safat, Kuwait; 3 Department of Oral Medicine and Radiology, Sharavathi Dental College and Hospital, Shivamogga, 577204 Karnataka India; 4 Department of Oral Medicine and Radiology, Faculty of Dentistry, Levy Mwanawasa Medical University (LMMU), Ministry of Health, Lusaka, Zambia

**Keywords:** Alpha amylase, Dental implant, Mechanical debridement, Mucin-4, Peri-implant mucositis, Whole saliva

## Abstract

**Background:**

The present study was based on the null hypothesis that there is no difference in clinicoradiographic parameters and whole salivary alpha amylase (AA) and mucin-4 levels before and after non-surgical mechanical debridement (NSMD) of patients with peri-implant mucositis (PM). The aim was to assess whole salivary AA and mucin-4 levels before and after treatment of PM.

**Methods:**

Patients with PM (Group-1) and individuals without peri-implant diseases (Group-2) were included. Demographic data was collected and peri-implant modified plaque and bleeding indices (mPI and mBI, respectively), probing depth (PD) and crestal bone loss were measured at baseline. Levels of AA and mucin-4 were assessed in unstimulated whole saliva samples. All patients underwent full-mouth non-surgical periodontal therapy (NSPT) and NSMD; and clinical parameters and salivary biomarkers were re-assessed after 3 months. Level of significance was set at *P* < 0.01.

**Results:**

Twenty-six and 32 individuals were included in groups 1 and 2, respectively. None of the participants had periodontitis. At baseline clinical periodontal parameters (PI [*P* < 0.001], GI [*P* < 0.001], clinical AL [*P* < 0.001] and PD [*P* < 0.001]) were significantly high in Group-1 than Group-2. At 3-month follow-up, there was a statistically significant reduction in clinical periodontal and peri-implant parameters (PI [*P* < 0.01], GI [*P* < 0.01], and PD [*P* < 0.01]) in Group-1 compared with their baseline values. At baseline, salivary AA levels were significantly high in Group-1 than Group-2 (*P* < 0.01). At 3-month follow-up, there was no significant difference in whole salivary AA levels among patients in groups 1 and 2.

**Conclusions:**

The AA and mucin-4 levels are potential biomarkers for evaluation of peri-implant diseases including PM. Mechanical instrumentation continues to be the most predictable treatment option for the management of peri-implant diseases.

## Introduction

Oral rehabilitation with dental implants is a modern alternate to traditional fixed and removable dental prostheses, such as bridges and dentures. Despite the fact that dental implants can osseointegrate and demonstrate success and survival rates of up to 100% [1, 2]; peri-implant diseases are a complication that cannot be overlooked. Peri-implant diseases initially manifest as peri-implant mucositis (PM) during which, gingival tissues appear red and puffy and demonstrate increased bleeding on gently probing (with or without suppuration) and probing depth (PD) [3, 4]. If PM is left undiagnosed and untreated for prolonged durations, the inflammatory process worsens and results in peri-implant crestal bone loss (CBL) or peri-implantitis [3, 4]. This may result in loosening of implant and even implant failure. The pathophysiology of peri-implant diseases is complex and driven by a cascade of events including immunoinflammatory and microbiological imbalances and genetic heterogeneity [5–9].

Studies [10, 11] have shown that patients with existing or with a history of periodontitis are more susceptible to peri-implant diseases compared with individuals with a healthy periodontal status. Unstimulated whole saliva (UWS) is a biologic oral fluid that expresses raised levels of destructive inflammatory cytokines among patients with periodontal and peri-implant diseases [12–16]. Alpha-amylase (AA) protein represents the autonomic nervous system and contributes towards maintenance of mucosal immunity by inhibiting bacterial colonization, reproduction and adhesion [17, 18]. Under oral inflammatory conditions, the production of AA is increased in an attempt to counteract the inflammatory insult [19]. It has been reported that a direct correlation exists between salivary AA activity and severity of periodontitis [20]. Similarly, mucins (primary organic constituent of mucus) envelop all mucosal surfaces of the body are glycoproteins that also contribute in innate immunity by facilitating bacterial clearance from the oral cavity [21]. In addition, mucins hydrate oral tissues thereby protecting oral hard and soft tissues from exogenous insults [22]. Furthermore, it has also been proposed such mucins act as receptors initiating intracellular signaling transduction pathways [23]. With reference to oral inflammatory conditions, Lundmark et al. [21] reported that mucin-4 is expressed in lower concentrations in UWS of saliva of patients with periodontitis compared with individuals with a healthy periodontal status. To date, there is one study [24] in indexed literature that has assessed salivary AA levels in relation to clinical implantology and associated research. However, this study [24] assessed salivary AA levels in relation to implant surgical interventions and not with reference to pathophysiology and/or severity of peri-implant diseases. It is worth mentioning that there are no clinical studies that have compared whole salivary mucin levels among patients with and without peri-implant diseases.

Non-surgical mechanical debridement (NSMD) is the gold standard for the management of PM and peri-implantitis [25, 26]. It is known that NSMD helps reduce the severity of clinical peri-implant inflammatory parameters (modified plaque index [mPI], modified bleeding index [mBI] and PD) in patients with peri-implant diseases [26, 27]. From a periodontal standpoint, non-surgical periodontal therapy has been reported to reduce salivary levels of destructive inflammatory cytokines, such as intrleukin-1beta (β) and matrix metalloproteinase (MMP)-8 [13]. Nevertheless, there are no studies in indexed literature that have compared whole salivary AA and mucin levels before and after NSMD in patients with PM. The aim was to assess whole salivary AA) and mucin-4 levels before and after NSMD of patients with PM. The present study was based on the *null* hypothesis that there is no difference in clinicoradiographic parameters and whole salivary AA and mucin levels before and after NSMD of patients with PM.

## Materials and methods

### Ethical standards

The present study was carried out in accordance with the Declaration of Helsinki as revised in 2013 guidelines involving human subjects. Volunteers were asked to sign a consent form. Withdraw did not bear any penalization or/and consequences. Ethical approval was obtained from ethics research committee of the Sharavathi Dental College and Hospital, Shivamogga, Karnataka, India (19/2022/CR). All individuals were also given verbal and written information about brushing and flossing techniques.

### Location and duration of study

The patients were recruited from the outpatient department at the College of Dentistry, Sharavathi Dental College and Hospital, Shivamogga, Karnataka 577204, India. The study was performed between August 2021 and March 2022. The co-author from this institution performed the clinicoradiographic investigations and laboratory-based investigations were done by a trained and calibrated technician.

### Inclusion and exclusion criteria

Patients having undergone dental implant therapy were included. Peri-implant mucositis was clinically defined as presence of peri-implant signs of soft-tissue swelling, redness and bleeding within 30 s of gently probing, and no additional CBL following initial healing [28]. Clinical definition of peri-implantitis was based on the following characteristics: (a) peri-implant signs of soft-tissue inflammation, (b) radiographic evidence of CBL after initial healing, and (c) increasing PD as compared to PD values recorded after prosthetic loading (PL) [28]. Peri-implant health was defined on the following features: absence of peri-implant clinical signs of soft-tissue inflammation, and absence of further CBL following PL [28]. In the present study, patients with a healthy periodontal and peri-implant status served as controls. Individuals habitually using combustible and non-combustible tobacco-products, habitual alcohol users, patients with existing or with a history of periodontitis and patients with self-reported systemic diseases such as prediabetes, diabetes mellitus (DM), cardiovascular diseases (CVD), obesity and oral and/or systemic cancer were excluded. Patients that had used antibiotics, bisphosphonates, probiotics, steroids and non-steroidal analgesics within the past 90 days were also excluded. Mandibular third molars, supernumerary teeth and broken-down teeth with embedded root remnants were not evaluated.

### Questionnaire

A questionnaire was used to gather information pertaining to patients’ age and gender, routine domestic oral hygiene maintenance (DOHM) protocols (brushing and interproximal flossing) and most recent visit to oral healthcare providers.

### Evaluation of patients’ dental records

Patients’ dental records were evaluated to gather the following information: number of implants per patient, implant dimensions (diameter and length), implant surface characteristics, implant loading protocol (delayed, immediate or early), implant abutment connection, implant jaw location, depth of placement (bone-level or submerged), duration in years for which, implants were in function and mode of prosthesis retention (cement and/or screw retention).

### Periodontal and peri-implant clinical and radiographic parameters

Full-mouth plaque index (PI) [29], gingival index (GI) [30], PD [31] and clinical attachment loss (AL) [32] were assessed around all teeth. Peri-implant mPI [33], mBI [33] and PD [34] were also measured. Clinical AL and PD were measured to the nearest millimeter (mm) with a plastic graded probe (Hu-Friedy InC, Chicago, IL, USA). Full-mouth digital intra-oral radiographs (Planmeca Romexis Intra oral X-Ray, Planmeca OY, Helsinki, Finland) were taken [35]; and standardization of all X-rays was done as described elsewhere [36, 37]. Marginal bone loss (MBL) and CBL around teeth and implants, respectively, were measured at baseline; and defined as linear distances from 2 mm below the cemento-enamel junction and implant abutment interface, respectively, to the alveolar crest [38, 39]. Clinical peri-implant and periodontal parameters were assessed at baseline and after 3 months of therapy.

### Non-surgical periodontal and peri-implant therapy

All patients underwent full-mouth non-surgical periodontal therapy (NSPT) and NSMD around teeth and implants, respectively. Sterile curettes (Gracey Curets, Hu-Friedy, Chicago, IL, USA) and an ultrasonic scaler (PIEZO-soft ultrasonic scaler; equipped with PIEZO Scaler tip 201, KaVo Dental, Germany) were used to perform NSPT. Peri-implant NSMD was done using sterile plastic curettes. Post-operatively, oral hygiene instructions were reinforced; and all patients were instructed to rinse every 12 h with 15 ml of 0.12% chlorhexidine gluconate for the next 14 days.

### Collection of whole saliva and assessment of alpha amylase and mucin levels

Collection of whole saliva samples was performed 24 h after clinical and radiographic evaluations. The protocol described by Ali et al. [35] was used to collect UWS samples. The whole salivary flow rate was determined. The UWS samples were collected during early morning hours (between 7 and 8am) with participants being in a fasting state. All saliva samples were immediately centrifuged at 1500×*g* for 15 min in a cold room; and the supernatants were stored at – 70 °C. All samples were assessed with 24 h of collection. The AA levels were as described by Haririan et al. [20]. In summary, whole salivary AA levels were determined using commercially available kits (Olympus-System-Reagents/6182, Olympus AU-640, Olympus Diagnostic Systems, PA, USA). A dilution protocol of 1:50 was used (assay range: 10–4800 U/L; intraassay CV < 1.4%; interassay CV < 3.3%). Whole salivary mucin-4 levels were measured as described by Lundmark et al. [21]. In summary, commercially available assay kits (Kamiya Biomedical Company, Seattle, WA, USA), were used according to the manufacturer’s instructions. The sensitivity for mucin-4 kits was 0.134 ng/ml, respectively. All samples were assessed by a trained and calibrated investigator (Kappa score 0.88).

### Sample-size estimation and statistical analyses

Data normality was assessed using the Kolmogorov–Smirnov test. Results of this test showed that all groups had a normality *P* value > 0.05 indicating normal distribution. Sample size was calculated to provide 95% power to recognize a significant difference of 2 mm among groups with a 95% confidence interval (α = 0.05); and assuming a standard deviation of 1 mm, considering the changes in mean PD. Therefore, at least 23 participants were required per group. Group comparisons were done using the paired *t* test. For multiple comparisons Bonferroni post-hoc adjustment test was performed. Correlation between demographic and clinicoradiographic parameters and whole salivary AA and mucin-4 levels were assessed using logistic regression models. When *P* values were less than 0.01, they are considered “statistically significant”.

## Results

### Participant screening

Initially, 96 patients with dental implants in function were invited to participate in the present study. Nineteen patients refused to sign the written informed consent form. On the remaining (*n* = 77), 14 patients were tobacco-smokers and five individuals had systemic diseases (three and two patients with DM and CVD, respectively). In total, 58 individuals (34 males and 24 females) signed the written informed consent form and were included in the present investigation.

### Demographics

Of the 58 individuals, 26 individuals (14 males and 12 females) had PM (Group-1) and the remaining (*n* = 32) had healthy peri-implant tissues (Group-2/controls). There was no statistically significant difference in the mean age of patients in groups 1 and 2. There was no significant difference in the mean age of males and females among patients with PM and controls. Tooth brushing twice daily was reported by 42.3% and 87.5% individuals with PM and controls, respectively. None of the patients with PM reported to have ever used a dental floss (Table 1).

**Table 1:** Characteristics of study groups

Parameters	Group-1	Group-2
Participants (*n*)	26	32
Mean age in years	44.8 ± 6.1 years	41.8 ± 1.4 years
Males	46.2 ± 3.5 years	43.5 ± 2.1 years
Females	42.5 ± 3.7 years	38.05 ± 0.8 years
Number of implants*	26 implants	32 implants
Maxilla	19 implants	21 implants
Mandible	7 implants	11 implants
Daily toothbrushing (twice)	11 (42.3%)	28 (87.5%)
Daily interproximal flossing	None	10 (31.3%)

*All implants were located in the region of missing premolars or molars

### Implants

A total of 58 implants were assessed. All implants were placed in the regions of missing premolars or molars. In groups 1 (*n* = 26) and 2 (*n* = 32), 19 and 21 implants, respectively, were located in the posterior maxilla (Table 1). All implants were placed at bone level, delayed-loaded and platform switched with moderately rough surfaces and had diameters and lengths ranging between 4 and 4.1 mm and 11 and 13 mm, respectively. Each implant was restored with a single ceramic crown using cement-retained restorations. In groups 1 and 2, implants were in function for a mean duration of 3.06 ± 0.2 and 3.4 ± 0.3 years, respectively.

### Periodontal and peri-implant clinicoradiographic status

At baseline clinical periodontal parameters (PI [*P* < 0.001], GI [*P* < 0.001], clinical AL [*P* < 0.001] and PD [*P* < 0.001]) were significantly high in Group-1 compared with Group-2. At 3-month, follow-up, there was a statistically significant reduction in clinical periodontal and peri-implant parameters (PI [*P* < 0.01], GI [*P* < 0.01], and PD [*P* < 0.01]) in Group-1 compared with their respective baseline values. None of the patients had active periodontal diseases, such as periodontitis. Clinical attachment levels, MBL and CBL did not demonstrate any significant difference when baseline values were compared with those measured at 3-month follow-up in both groups. In Group-2, there was no significant difference in periodontal and peri-implant clinicoradiographic status at both time intervals (Table 2).

**Table 2:** Periodontal and peri-implant status among patients with peri-implant mucositis and controls at baseline and 3-month follow-up

Parameters	Baseline		3-month follow-up	
	Group-1	Group-2	Group-1	Group-2
Full-mouth periodontal status				
Plaque index	0.77 ± 0.003*^†^	0.2 ± 0.004	0.23 ± 0.005	0.15 ± 0.003
Bleeding index	0.85 ± 0.005*^†^	0.3 ± 0.004	0.28 ± 0.1	0.31 ± 0.08
Probing depth	4.4 ± 0.2 mm*^†^	1.8 ± 0.2 mm	1.6 ± 0.04 mm	1.8 ± 0.08 mm
Clinical attachment loss	2.05 ± 0.04 mm*	0.7 ± 0.007 mm	2 ± 0.007 mm	0.7 ± 0.005 mm
Marginal bone loss	2.1 ± 0.3 mm*	0.8 ± 0.05 mm	2.5 ± 0.2 mm	0.6 ± 0.03 mm
Peri-implant status				
Modified plaque index	0.82 ± 0.1*^†^	0.1 ± 0.0004	0.2 ± 0.02	0.1 ± 0.003
Modified gingival index	0.86 ± 0.08*^†^	0.2 ± 0.0007	0.13 ± 0.002	0.1 ± 0.002
Probing depth	4.3 ± 0.1 mm*^†^	1.5 ± 0.03 mm	1.9 ± 0.04 mm	1.4 ± 0.02 mm
Crestal bone loss	0.7 ± 0.3 mm*	0.4 ± 0.0005 mm	0.7 ± 0.2 mm	0.4 ± 0.003 mm

*Compared with Group-2 at baseline (*P* < 0.01) using the paired *t* test

^†^Compared with Groups-1 (*P* < 0.001) and 2 (*P* < 0.01) at 3-month follow-up using the paired *t* test and the Bonferroni Post-hoc test

### Salivary flow rate and whole salivary alpha amylase and mucin-4 levels

There was no significant difference in SFR among patients in groups 1 and 2 throughout the study period. At baseline, salivary AA levels were significantly high in Group-1 compared with Group-2 (*P* < 0.01). At 3-month follow-up, there was no significant difference in whole salivary AA levels among patients in groups 1 and 2. Salivary mucin-4 levels were significantly high in Group-2 compared with Group-1 at baseline (*P* < 0.01). There was no significant difference in whole salivary mucin-4 levels among patients in groups 1 and 2 at 3-month follow-up (Table 3).

**Table 3:** Periodontal and peri-implant status among patients with peri-implant mucositis and controls at baseline and 3-month follow-up

Parameters	Baseline		3-month follow-up	
	Group-1	Group-2	Group-1	Group-2
Salivary flow rate	0.12 ± 0.06 ml/min	0.11 ± 0.02 ml/min	0.13 ± 0.01 ml/min	0.12 ± 0.01 ml/min
Salivary AA levels (U/L)	209.6 ± 26.5 U/L*^†^	25.6 ± 4.1 U/L	26.8 ± 10.2 U/L	20.3 ± 6.1 U/L
Mucin-4 levels (ng/ml)	0.51 ± 0.08 ng/ml*^†^	1.22 ± 0.06 ng/ml	1.04 ± 0.06 ng/ml	1.26 ± 0.04 ng/ml

*Compared with Group-2 at baseline (*P* < 0.01) using the paired *t* test

^†^Compared with Groups-1 (*P* < 0.01) and 2 (*P* < 0.01) at 3-month follow-up using the paired *t* test and the Bonferroni Post-hoc test

### Correlation between whole salivary alpha amylase and mucin-4 levels and demographic and clinicoradiographic parameters

**Fig. 1:**
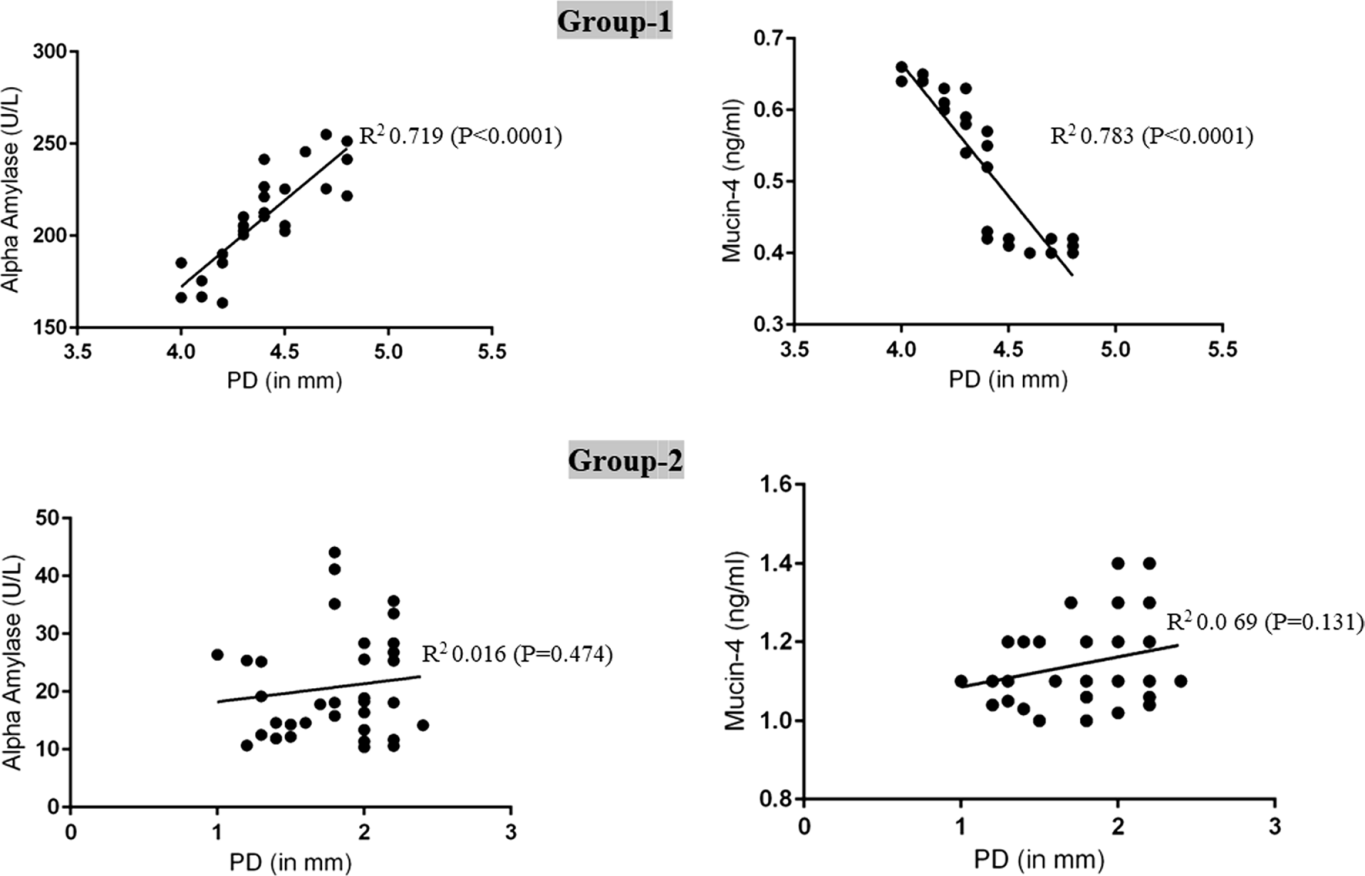
Logistic regression analysis for the correlation between whole salivary alpha amylase and mucin-4 levels and peri-implant probing depth in groups 1 and 2 at baseline

In Group-1, there was a statistically significant correlation between peri-implant PD and whole salivary AA and mucin-4 levels at baseline (Fig. 1). There was no statistically significant correlation between peri-implant PD and whole salivary AA and mucin-4 levels at baseline in Group-2 (Fig. 1). At 3-month follow-up, there was no statistically significant correlation between periodontal and peri-implant clinicoradiographic parameters in groups 1 and 2 (data not shown). There was no statistically significant correlation between age, gender, years of implants in function, and whole salivary AA and mucin-4 levels in both groups (data not shown).

## Discussion

The present study was based on the *null* hypothesis that there is no difference in clinicoradiographic parameters and whole salivary AA and mucin levels before and after NSMD of patients with PM. To date, there is one study [24] in indexed literature that has assessed salivary AA levels in relation to dental implants. Similarly, there are no studies that have correlated whole salivary mucin-4 levels with peri-implant diseases. Sabbagh et al. [24] assessed fluctuations in salivary AA levels during surgical placement of dental implants and correlated it with patients’ heart rate. Laboratory-based results of the present investigation correlated whole salivary AA and mucin-4 levels in patients with and without PM. Our results showed that whole salivary mucin-4 and AA levels were significantly lower and higher, respectively, in patients with PM and controls. Lundmark et al. [21] reported significantly lower salivary mucin-4 levels in patients with periodontitis. The authors support the results reported by Lundmark et al. [21] as the current results showed significantly lower mucin-4 levels in Group-1 (patients with PM) than controls (patients without peri-implant diseases). There is a likelihood that salivary AA and mucin-4 levels significantly altered in patients with peri-implantitis than those with PM; however, this evaluation could not be performed in the current investigation. A variety of salivary markers including interleukin-1 beta, tumor necrosis factor-alpha, and soluble urokinase plasminogen activating receptor have been suggested to play a role in the etiopathogenesis and progression of periodontal and peri-implant diseases [16, 35, 40, 41]; however, a consensus on the most reliable and predictable salivary biomarker peri-implant disease activity is yet to be reached. Nevertheless, results based on logistic regression analysis showed a significant correlation between baseline peri-implant PD and salivary AA and mucin-4 levels in patients with PM. Based upon the present results, whole salivary AA and mucin-4 levels can also be considered as potential biomarkers of peri-implant disease activity.

As far as controls (Group-2) are concerned, these individuals had healthy implants (defined according to criteria reported elsewhere [28]); and were visiting the oral healthcare facility for routine dental prophylactic/hygiene maintenance. Studies [42, 43] have shown that patients with a history of periodontitis are at an increased risk of developing peri-implant diseases, such as peri-implantitis compared with individuals with a healthy periodontal health status. Despite the fact that none of the participants included in Group-1 had existing or a history of periodontitis, peri-implant diseases (PM) occurred in these individuals. One reasoning for this is that DOHM protocols appeared compromised in these individuals. As shown in Table 1, approximately 42% individuals in Group-1 reported that they were brushing teeth twice daily compared with individuals with “healthy implants”/controls, where approximately 87% individuals reported that they were brushing twice daily. Moreover, flossing of interproximal spaces was being routinely performed by nearly 10% patients in the control group, whereas none of the individuals in Group-1 reported to have ever used a dental floss. These are potential factors that may have facilitated the accumulation of supra- and subgingival plaque accumulation around periodontal and peri-implant tissues thereby reflecting significantly higher baseline scores of PI, mPI, GI, mGI, and clinical AL among patients in Group-1 compared with Group-2. Interestingly, the mean crestal and MBL was less than 3 mm in both groups at baseline despite routine OHM protocols being compromised among patients in group 1 than Group-2. By no means should these outcomes suggest that toothbrushing once daily and lack of flossing sufficient for routine DOHM. However, there are certain factors that could be used to explain this finding. In the present investigation, all participants were relatively young with a mean age of approximately 40 years (Table 1). It has been reported that periodontitis is more often manifested in older patients (aged at least 60 years and above) compared with individuals that are in their fourth decade of life [38]. This may be a risk-factor of peri-implantitis as well; however, there is no consensus in this regard. In the present study, all implants were in function for a mean duration of approximately 3 years, which is a relatively short duration. Studies [2, 44, 45] have shown that under physiologic conditions dental implants demonstrate minimal CBL up to 7 years of PL. The authors applaud results reported in a non-interventional multicenter study [45] according to which, dental implants placed in individuals (mean age approximately 50 years) demonstrate CBL of 0.52 ± 0.55 mm after 36 months of PL. In the current investigation, the CBL values at 3 years of PL were approximately 0.7 mm and 0.4 mm, respectively, in groups 1 and 2. It is, therefore, speculated that severity of peri-implant diseases is worse and salivary mucin-4 and AA levels are markedly altered in elderly individuals with a duration of implant PL (over 10 years) compared with younger individuals with a shorter duration (≤ 5 years) of implant PL. Further studies are needed to test this hypothesis.

Non-surgical debridement of periodontal and peri-implant surfaces and tissues is classically performed for the management of periodontal and peri-implant diseases [27]. However, the contribution of improvements in oral hygiene maintenance protocols cannot be ignored. There is a possibility that the participants, especially those in Group-1 improved their DOHM protocols after therapeutic interventions. However, an oral health questionnaire was not administered to all participants at the follow-up visit.

Although stringent eligibility criteria were imposed for patient inclusion to minimize the risk of bias and standardize the study groups; this may be considered as a potential limitation of the present study. Studies [46, 47] have reported that habitual use of nicotinic products (such as cigarettes, waterpipe and electronic nicotine delivery systems) and a compromised medical health status (such as poor glycemic control) are risk factors of periodontal and peri-implant diseases (PM and peri-implantitis). It is tempting to speculate that salivary AA and mucin-4 levels are significantly altered in diabetic smokers compared with systemically healthy smokers and never-smokers. However, despite the fact that CBL is higher in smokers than never-smokers, there is insufficient evidence in indexed literature to nominate smoking as a risk-factor of peri-implantitis [48]. Likewise, results from a narrative review [48] reported that patients with DM diagnosed with peri-implant mucositis are not at a higher risk to develop peri-implantitis when compared to systemically healthy controls. Therefore, it is challenging to declare that smoking and DM are “risk-factors” of peri-implant diseases. It is also noteworthy that there were a limited number of implants assessed in the present study and that all patients had a healthy periodontal status. It is, therefore, speculated that the AA and mucin-4 levels reported in the present study could be indicative of a healthy periodontal status. Furthermore, due to the ethically approved study design and limitations in funding resources, other laboratory-based investigations such as assessment of whole salivary total protein concentrations and assessment of microbes in the subgingival oral biofilm before and after NSPT and NSMD were not done. This warrants further studies with specific objectives.

## Conclusions

The AA and mucin-4 levels are potential biomarkers for evaluation of peri-implant diseases including PM. Mechanical instrumentation continues to be the most predictable treatment option for the management of peri-implant diseases.

## Data Availability

The data are available from the corresponding author on reasonable request.

## Abbreviations

AA: Alpha amylase
CBL: Crestal bone loss
DOHM: Domestic oral hygiene maintenance
GI: Gingival index
mPI: Modified plaque index
mBI: Modified bleeding index
NSMD: Non-surgical mechanical debridement
NSPT: Non-surgical periodontal therapy
PD: Probing depth
PI: Plaque index
PM: Peri-implant mucositis
UWS: Unstimulated whole saliva
